# Hydrated proton complexes supplementation for tumor microenvironment reprogramming: a bioenergetic strategy targeting the Warburg effect and mitochondrial dysfunction

**DOI:** 10.3389/fonc.2025.1647054

**Published:** 2025-09-05

**Authors:** Alfred Lee Edgar, Luis Felipe Dias Lopes, Eduarda Grando Lopes, Izabella Danezi Felin, Carlos Roberto Felin, João Francisco Pollo Gaspary

**Affiliations:** ^1^ Research and Development Department, ElastroCrete, LLC, Veyo, UT, United States; ^2^ Department of Administrative Sciences, Federal University of Santa Maria, Santa Maria, Brazil; ^3^ Veterinary Medicine Course, Federal University of Santa Maria, Santa Maria, Brazil; ^4^ Department of Pathology, Federal University of Santa Maria, Santa Maria, Brazil; ^5^ Oncocenter Clinic, Santa Maria, Brazil; ^6^ Instituto AuBento – Center for Teaching, Clinical Practice, and Research in Orthomolecular and Translational Health Innovation, Santa Maria, Brazil

**Keywords:** tumor microenvironment, hydrated proton complexes, mitochondrial dysfunction, Warburg effect, redox signaling, bioenergetic reprogramming

## Abstract

**Background:**

The tumor microenvironment (TME) is characterized by a reversed pH gradient—acidic extracellular and alkaline intracellular conditions—arising from mitochondrial dysfunction, metabolic reprogramming, and dysregulated proton transport. These alterations establish a permissive niche for tumor progression, immune evasion, and resistance to therapy. Although the TME is increasingly recognized as a key determinant of cancer behavior, effective and targeted strategies for its bioenergetic reprogramming remain scarce.

**Objectives:**

This study introduces and evaluates Eigen/Zundel Complexes-Rich Water (EZC-Rich Water) as a novel hydrated proton supplementation strategy capable of targeting Warburg-induced proton dysregulation and restoring mitochondrial function, while stabilizing electrochemical membrane dynamics within the TME.

**Methods:**

A structured translational research design was implemented, combining Work Breakdown Structure (WBS), Open Innovation, and Design Thinking methodologies. This approach enabled the identification of Fundamental Points of View (FPV’s)—physiological targets underlying TME dysfunction—and Critical Success Factors (CSF’s)—mechanistic requirements for therapeutic efficacy. Multicriteria decision analysis was applied to integrate findings from oncology, bioenergetics, and physical chemistry, linking hydrated proton supplementation to improved zeta potential, electrosmotic flow, mitochondrial coupling, and redox regulation.

**Results:**

Integrative analyses demonstrated that EZC-Rich Water delivers metastable hydrated proton clusters (H_9_O_4_^+^ and H_5_O_2_^+^) that support selective and efficient proton transfer via the Grotthuss mechanism. This supplementation facilitates compartmentalized acid–base modulation without inducing systemic acidosis, aligning with prioritized FPV’s and validated CSF’s. The proposed strategy shows translational potential to correct pH inversion, optimize oxidative phosphorylation, and restore bioenergetic integrity in the TME.

**Conclusion:**

Hydrated proton supplementation through EZC-Rich Water represents an innovative bioenergetic intervention with potential to reprogram the tumor microenvironment. By targeting core metabolic dysfunctions such as the Warburg effect and mitochondrial uncoupling, this clinically adaptable and low-risk strategy introduces a new paradigm in nutritional oncology. Further preclinical and clinical studies are warranted to validate its efficacy, safety, and translational applicability in oncology and related metabolic disorders.

**Systematic review registration:**

https://www.crd.york.ac.uk/prospero/display_record.php?, identifier CRD420251065137; https://www.crd.york.ac.uk/prospero/display_record.php?, identifier CRD420251022205.

## 1 Introduction

The tumor microenvironment (TME) represents a highly dynamic and pathophysiologically distinct compartment that sustains cancer progression through metabolic reprogramming, immune evasion, and resistance to therapy (1, 2). A defining hallmark of the TME is the inversion of the normal acid-base gradient, resulting in acidic extracellular pH (pHe) and alkaline intracellular pH (pHi) (3, 4). This dysregulated pH landscape enhances glycolytic flux (the Warburg effect)—a metabolic reprogramming whereby cancer cells preferentially convert glucose to lactate via aerobic glycolysis even in the presence of sufficient oxygen, diverting pyruvate away from mitochondrial oxidative phosphorylation. This shift supports anabolic growth, contributes to extracellular acidification, and promotes invasive phenotypes, while also impairing drug delivery by altering transmembrane electrochemical potentials and protonation-dependent drug activity (5–8).

Cancer cells achieve this pH inversion through coordinated upregulation of proton pumps (H^+^-ATPases), Na^+^/H^+^ exchangers, and carbonic anhydrases—particularly CAIX under hypoxic conditions (9–14). These adaptations are intimately linked to mitochondrial dysfunction, which disrupts proton-coupled oxidative phosphorylation, impairs redox balance, and further reinforces extracellular acidification via lactate and proton export (15–17). The resulting bioenergetic instability not only compromises the effectiveness of conventional therapies but also fosters a microenvironment that is inherently resistant to immune and pharmacological interventions (1, 4, 6, 8).

Despite advances in molecular oncology, clinically effective and targeted strategies for microenvironmental modulation remain limited, especially in the face of therapeutic resistance and metabolic plasticity (18–21). This translational gap underscores the urgent need for innovative approaches capable of restoring acid-base and redox homeostasis within the TME—a recognized driver of tumor progression and poor therapeutic response.

In a previous study, Gaspary et al. (2024) (15) demonstrated that controlled, hormetic administration of carbon dioxide could partially restore mitochondrial and acid-base homeostasis by enhancing carbonic acid availability and modulating proton gradients. These findings provided translational insights into employing physicochemical agents to modulate the biophysical parameters of the tumor microenvironment (TME), thereby opening novel investigative pathways for adjunctive cancer therapies focused on targeted pH regulation.

Building on these translational foundations, the present study introduces Eigen/Zundel Complexes-Rich Water (EZC-Rich Water) as a next-generation nutritional intervention, conceptually developed through a structured innovation framework integrating Open Innovation (22), Design Thinking (23), and multicriteria analysis (24). EZC-Rich Water is designed to modulate the tumor microenvironment by delivering bioavailable hydrated proton complexes—Eigen (H_9_O_4_^+^) and Zundel (H_5_O_2_^+^) (25)—which play central roles in proton transport through the Grotthuss mechanism, supporting localized acid-base modulation and enhancing mitochondrial proton motive force (26, 27). In contrast to conventional acidified solutions, EZC-Rich Water offers a structured and targeted proton delivery system, minimizing disruption to systemic electrolyte balance and reducing the risk of cytotoxic acidification (25, 28, 29).

This paper presents a structured translational synthesis of bioenergetic evidence supporting the use of EZC-Rich Water to reprogram the TME, restore mitochondrial efficiency, optimize redox potential, and stabilize cellular membrane dynamics. By bridging fundamental proton chemistry with cancer metabolism in a systematically integrated framework, we propose hydrated proton supplementation as a novel, clinically feasible, and mechanistically sophisticated adjunct to contemporary oncological strategies. This approach is positioned to address both the biochemical vulnerabilities of the TME and the pressing translational need for innovation in cancer therapy. This approach directly targets the metabolic signature of cancer, notably the Warburg effect and mitochondrial dysfunction, offering a promising pathway for TME reprogramming.

## 2 Methods

This study followed a translational research design structured through the Work Breakdown Structure (WBS) methodology (30, 31), allowing systematic decomposition of the research question into interdependent and chronologically ordered Work Packages (WP’s). Each WP was conceived to explore a specific layer of evidence required to assess the bioenergetic and clinical feasibility of hydrated proton supplementation with Eigen/Zundel Complexes-Rich Water (EZC-Rich Water) in modulating tumor microenvironment dynamics. The WBS framework was supported by principles of Open Innovation (22) and Design Thinking (23), facilitating conceptual triangulation and hypothesis refinement across bioenergetic, metabolic, and oncological domains. The research project was organized into six interconnected WPs, as summarized below.

### 2.1 WP1 – strategic project governance and methodological oversight

**Figure 1:**
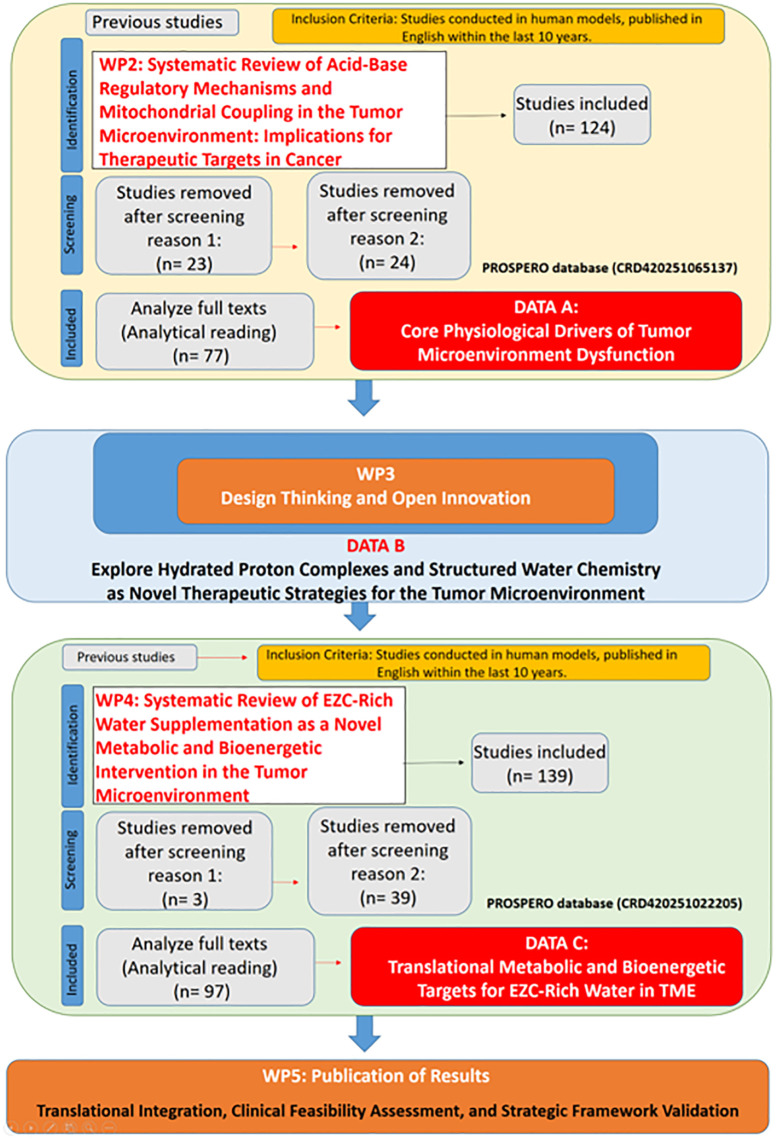
Structured methodological framework for translational research on hydrated proton complexes and bioenergetic modulation in the tumor microenvironment.

This initial work package ensured alignment with translational standards and methodological consistency across all stages of the research. A supervisory panel with expertise in bioenergetics, oncology, and biomedical engineering monitored the implementation of each WP, ensuring compliance with PRISMA guidelines in systematic reviews (32) and maintaining documentation according to PROSPERO standards where applicable. Iterative review cycles enabled adjustments in the scope and terminology across WPs as new evidence emerged. The structured methodological approach, outlining the sequential and integrative nature of all Work Packages (WP’s), is depicted in **Figure 1**.

### 2.2 WP2 – systematic review of electrochemical and metabolic regulation in the tumor microenvironment: implications for therapeutic targeting

**Figure 2:**
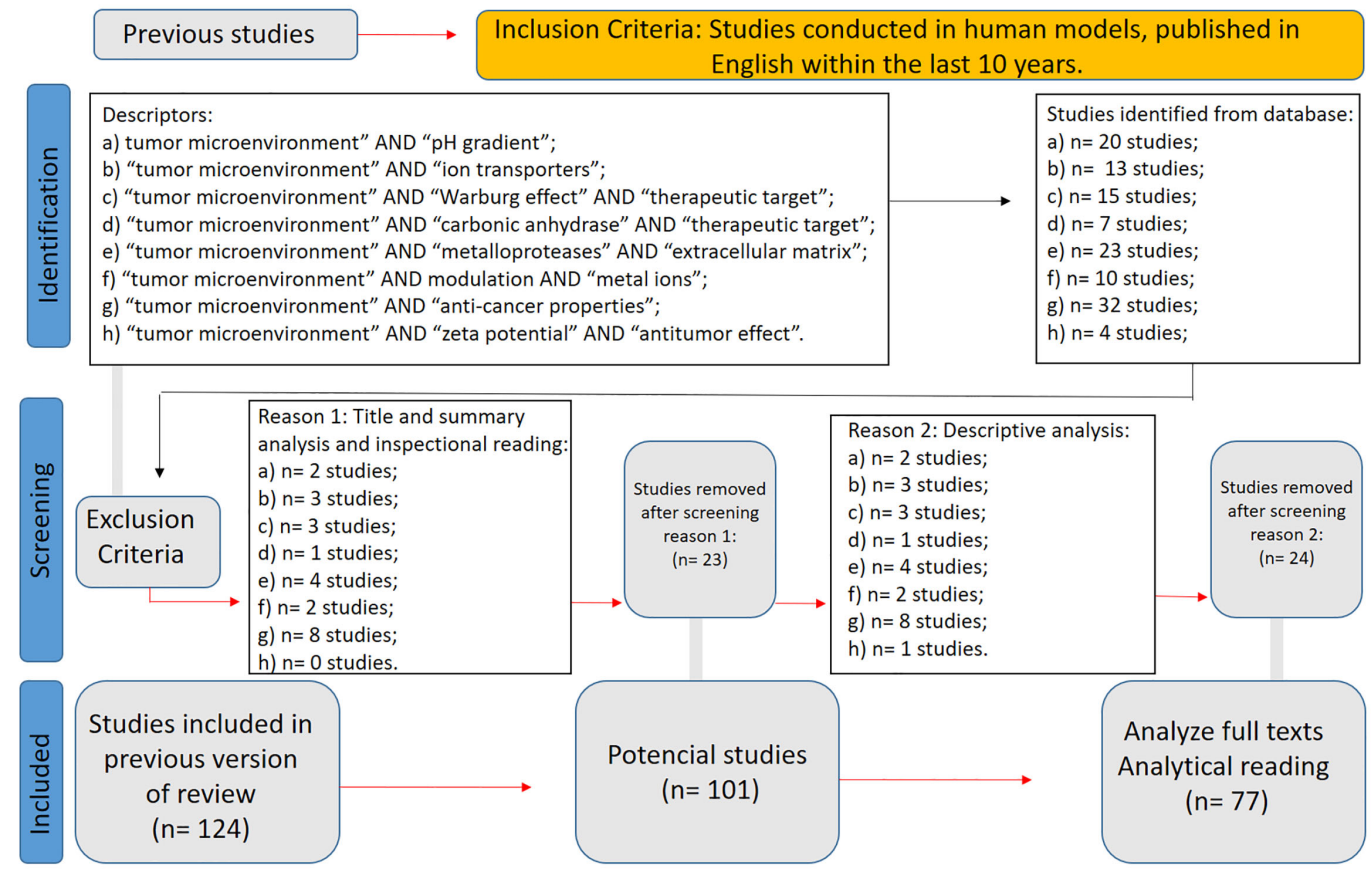
WP2 PRISMA flow diagram.

A targeted systematic review was conducted and formally registered in the PROSPERO database (CRD420251065137) to map the core physiological mechanisms underlying pH inversion and bioenergetic dysregulation within the tumor microenvironment (TME), emphasizing intracellular alkalinization, extracellular acidification, mitochondrial coupling failure, and metal ion-related modulation. Searches were performed in major databases using the following indexed terms: “tumor microenvironment” AND “pH gradient” (20 results); “tumor microenvironment” AND “ion transporters” (13 results); “tumor microenvironment” AND “Warburg effect” AND “therapeutic target” (15 results); “tumor microenvironment” AND “carbonic anhydrase” AND “therapeutic target” (7 results); “tumor microenvironment” AND “metalloproteases” AND “extracellular matrix” (23 results); “tumor microenvironment” AND modulation AND “metal ions” (10 results); “tumor microenvironment” AND “anti-cancer properties” (32 results); and “tumor microenvironment” AND “zeta potential” AND “antitumor effect” (4 results). Filters applied included studies conducted in human models, published in English within the last 10 years. After title and abstract screening, a total of 124 studies were identified. Following full-text review and methodological assessment, 77 studies were ultimately included, prioritizing mechanistic, physiological, and translational findings (**Figure 2**).

Based on the findings from WP2, this stage applied a multicriteria analysis adapted from the decision-support framework proposed by Bana e Costa et al. (1999) (24) to systematically identify Fundamental Points of View (FPV’s), representing essential physiological targets relevant for innovative cancer interventions, and Critical Success Factors (CSF’s), defined as mechanistic drivers crucial for effectively modulating tumor pH and mitochondrial bioenergetics. These FPV’s and CSF’s were further refined through an integrative literature review encompassing studies on mitochondrial membrane potential, zeta potential, intracellular redox regulation, and proton-coupled transport mechanisms.

### 2.3 WP3 – design thinking and open innovation for hydrated proton complexes and structured water chemistry as novel therapeutic strategies for the tumor microenvironment

Based on the multicriteria analysis data provided by WP2, WP3 was structured to systematically explore novel translational opportunities by integrating Open Innovation (22) and Design Thinking (23) frameworks. This WP applied a structured methodological framework to systematically explore the therapeutic potential of hydrated proton complexes, specifically Eigen and Zundel configurations, and structured water chemistry as novel modulators of the tumor microenvironment (TME). An in-depth scientific and translational review was conducted to elucidate the role of these complexes in enhancing hydrated proton mobility via the Grotthuss mechanism, emphasizing their distinct physicochemical characteristics compared to conventional acidified water. Key molecular properties, including proton hopping efficiency, electroconductivity, stability of the zeta potential, and interactions at biological membrane interfaces, were critically analyzed to assess the viability of their therapeutic application through structured water supplementation. Experimental insights were carefully drawn from recent advancements in physical chemistry, electrochemistry, dielectric biology, and translational biomedical research.

The translational rationale developed through WP3 informed a subsequent targeted systematic review (WP4) to assess specifically the bioenergetic and metabolic evidence supporting EZC-Rich Water as an innovative nutritional adjunct in oncology.

### 2.4 WP4 – systematic review of EZC-Rich water supplementation as a metabolic and bioenergetic intervention in the tumor microenvironment

A second systematic review was registered in the PROSPERO database (CRD420251022205) to assess translational evidence supporting the use of structured water enriched with Eigen/Zundel complexes as a nutritional strategy for human health optimization. Indexed descriptors included “hydronium”, “structured water”, “hydrated proton”, “zeta potential”, and “bioenergetics”. Inclusion criteria prioritized peer-reviewed human or *in vitro* studies reporting effects on mitochondrial function, pH modulation, membrane permeability, and systemic redox balance.

WP5 was designed to systematically investigate the translational relevance of structured water supplementation enriched with hydrated proton complexes—specifically Eigen (H_9_O_4_^+^) and Zundel (H_5_O_2_^+^) structures—focusing on their capacity to modulate human bioenergetic, membrane electrochemical, and acid-base dynamics. A systematic literature review was conducted and registered under the PROSPERO database (CRD420251022205), following PRISMA guidelines (32).

The search strategy targeted descriptors such as “hydronium,” “Eigen complex,” “Zundel complex,” “hydrated proton,” “structured water,” “proton mobility,” and “zeta potential,” across PubMed, Scopus, and LILACS databases. Studies were included if they reported effects on pH modulation, mitochondrial function, membrane permeability, electrosmotic dynamics, or systemic bioenergetic regulation. Only peer-reviewed articles in English published after 2004 were considered.

**Figure 3:**
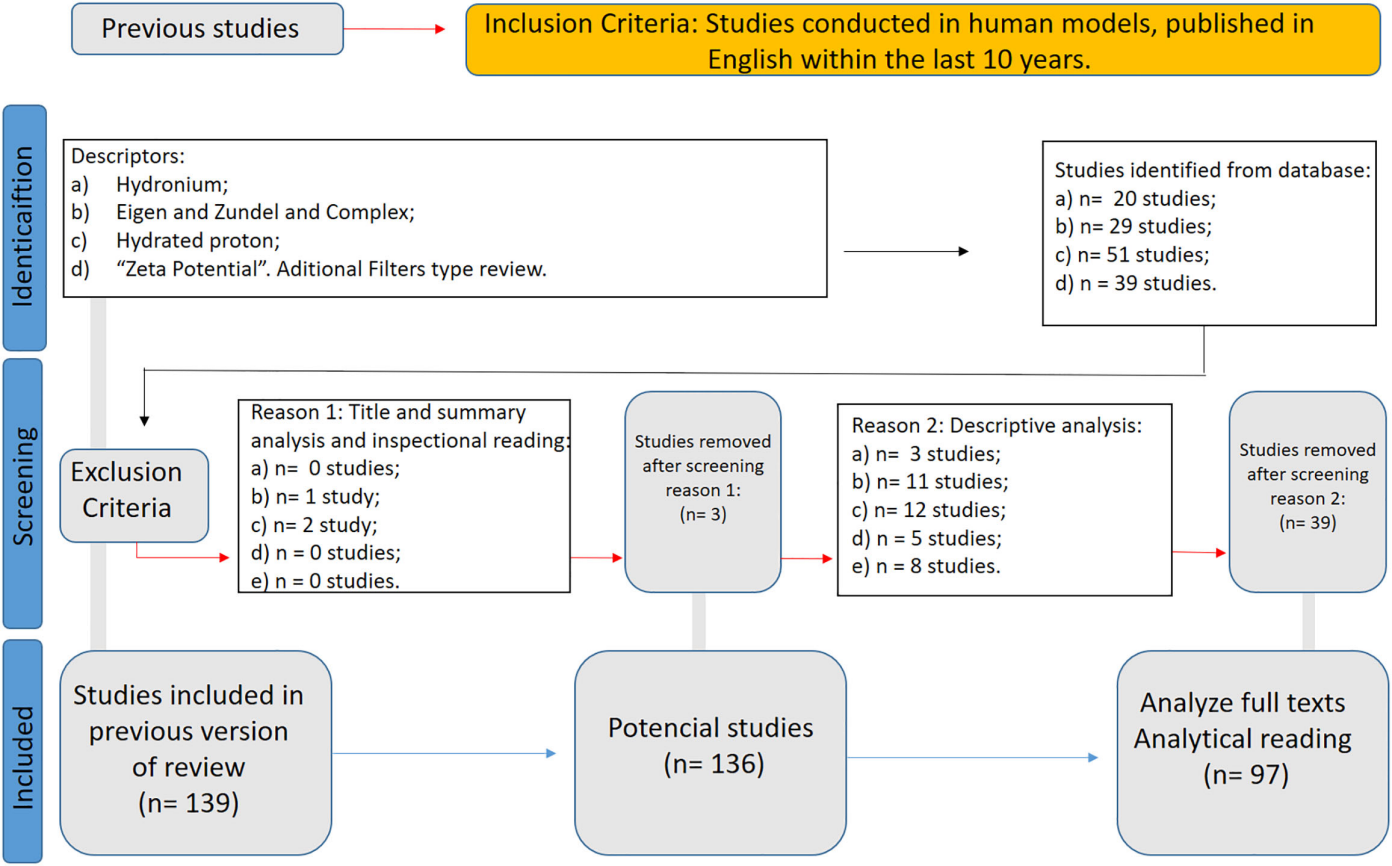
WP4 PRISMA flow diagram.

From an initial pool of 139 identified records, 42 studies were excluded after title and abstract screening. Of the 97 articles subjected to full-text review, all were retained for final qualitative synthesis. The detailed literature selection process for this systematic review, conducted according to PRISMA guidelines, is illustrated in **Figure 3**.

Finally, the comprehensive data and insights obtained from WP2 to WP4 were synthesized and critically evaluated in WP5, establishing a robust translational foundation for future experimental validation and publication.

### 2.5 WP5 – translational integration, clinical feasibility assessment, and strategic framework validation

This final stage synthesized and integrated findings from all previous WPs, employing structured methodological frameworks including SMART (Specific, Measurable, Achievable, Relevant, Time-bound) (33) and FINER (Feasible, Interesting, Novel, Ethical, Relevant) criteria (34). The therapeutic and translational potential of EZC-Rich Water was systematically assessed as a hydrated proton supplementation strategy for adjunctive cancer treatment, evaluating hypothesis plausibility, potential routes of administration, biomarker targets (e.g., zeta potential modulation, mitochondrial efficiency, redox homeostasis), and safety considerations. Concrete translational objectives, measurable biochemical and physiological outcomes, and the technological achievability of proposed interventions were critically analyzed. Relevance to current unmet needs in nutritional and metabolic oncology was highlighted, as was the novelty and ethical viability of the proposed supplementation strategy. Finally, this WP provided clear recommendations for future experimental validation (*in vitro*, *in vivo*, clinical trials) and structured a robust scientific rationale suitable for peer-reviewed publications.

## 3 Results

The Work Breakdown Structure (WBS) methodology (30, 31) allowed for the progressive consolidation of translational evidence across six interconnected Work Packages (WP2 to WP5). This structure enabled the integration of findings from oncology, bioenergetics, and water chemistry, guiding the formulation of a coherent hypothesis: that supplementation with water enriched in hydrated proton complexes—specifically Eigen and Zundel structures—may serve as a strategic adjunct for modulating tumor microenvironment dynamics, particularly through pH gradient correction, mitochondrial optimization, and redox regulation.

Each WP generated distinct insights that collectively reinforce the biological plausibility and therapeutic potential of EZC-Rich Water in the context of cancer bioenergetics. The sections below detail the findings from each WP sequentially.

### 3.1 WP2 – mechanistic drivers of electrochemical and bioenergetic imbalance in the tumor microenvironment

The WP2 comprised a targeted systematic review designed to elucidate the primary physiological and molecular mechanisms underpinning acid-base dysregulation within the tumor microenvironment (TME). These studies collectively underscored the presence of a reversed pH gradient as a hallmark of cancerous tissues. Specifically, extracellular pH (pHe) typically ranged between 6.2 and 6.9, contrasting markedly with the alkaline intracellular pH (pHi) values observed between 7.12 and 7.65. This inversion contrasts sharply with physiological norms in healthy tissues, where intracellular pH ranges from 7.0 to 7.2, and extracellular pH from 7.3 to 7.4. Such pathological acidification of the extracellular milieu is closely associated with tumor invasiveness, apoptosis resistance, therapeutic failure, genomic instability, and immune evasion (1, 3, 4, 35–37).

Central mechanisms identified as driving this abnormal pH landscape include the upregulation of proton transporters such as H^+^-ATPases, carbonic anhydrase IX (CAIX) (9–11), and Na^+^/H^+^ exchangers (37–39), alongside metabolic adaptations exemplified by the Warburg effect (19, 20), promoting aerobic glycolysis and subsequent lactic acid production. Furthermore, conditions of hypoxia (15), inflammatory signaling cascades, and impaired mitochondrial oxidative phosphorylation (16–18) were consistently highlighted as upstream modulators reinforcing these disturbances and driving the reversal of the pH gradient (40).

Critically, heterogeneity in pH regulation emerged as a recurring theme, with numerous studies documenting pronounced inter- and intra-tumoral variability. This variability, characterized by distinct microregional pH profiles, was posited as contributing significantly to tumor aggressiveness and therapeutic resistance (40–42).

Based on this comprehensive synthesis, a structured set of Fundamental Points of View (FPV’s) was formalized to encapsulate the principal acid-base and bioenergetic abnormalities characteristic of the TME (**Table 1**). The final structure of the Fundamental Points of View (FPV’s) emerged from an iterative and integrative synthesis of recent systematic reviews, experimental studies, and high-impact position papers on the tumor microenvironment. Each FPV represents a critical and translationally actionable axis identified across multiple literature filters and thematic searches.

**Table 1:** Fundamental Points of View (FPV’s) associated with tumor microenvironment dysregulation.

FPV	Description	Key References and Filters
1. Reversed pH Gradient	Pathological inversion of acid-base balance, characterized by extracellular acidification and intracellular alkalinization, driving invasiveness, immune evasion, drug resistance, and genomic instability.	Persi et al. (2018); Shirmanova et al. (2015); Zheng et al. (2020); Gong et al. (2025); Piasentin et al. (2020); (3, 4, 40, 43, 44)
2. Dysregulated Proton/Ion Transport	Imbalance in proton and ion (H^+^, Na^+^, K^+^) homeostasis perpetuating pathological gradients, contributing to tumor invasion, metastasis, and TME remodeling.	Boedtkjer (2022); Alfarouk, 2016; Gentile et al. (2025); Peretti et al. (2019) Cardone et al. (2023) (35, 45–48)
3. Mitochondrial Coupling Dysfunction	Aberrant mitochondrial function leading to excessive ROS production, bioenergetic dysfunction, and metabolic plasticity underpinning tumor progression.	Li et al. (2024); Di Gregorio et al. (2022); Wang et al. (2023); Cardone et al. (2019); Cardone et al. (2023) (16–18, 48, 49)
4. Membrane Electrochemical Instability	Disruption of electrochemical gradients impairing cellular signaling, metabolism, and matrix interactions, promoting malignancy and therapy resistance.	Fecikova et al. (2024); Miranda-Gonçalves et al. (2016); Schniers et al. (2021); Kim et al. (2025) (50–53)
5. Metal Ion Homeostasis and Copper Metabolism Dysregulation	Dysregulated metal ion (Cu, Fe, Zn, Mn) metabolism central to tumor biology, driving angiogenesis, matrix remodeling, regulated cell death, immune modulation, and metabolic reprogramming. Emerging evidence positions copper dysregulation as a novel and actionable vulnerability (cuproplasia/cuproptosis).	Che et al. (2024); Lu et al. (2025); Pang (2025); Lin et al. (2023); Wang et al. (2023); Kong et al. (2024) (54–59)

Subsequent systematic searches (totaling 77 additional studies) robustly validated these FPV’s, particularly reinforcing the centrality of reversed pH gradients, mitochondrial dysfunction, dysregulated ion transport, and copper metabolism dysregulation. Although no additional FPV’s emerged, recent literature emphasized novel downstream effectors, such as the pH-sensitive receptor GPR4 (60) and the ion channel TRPC4 (61), further underscoring the complexity and potential therapeutic opportunities inherent within the acid-base and metabolic landscape of tumors.

Targeted analyses using specific descriptors further strengthened these conclusions. Studies filtered under “tumor microenvironment” and “ion transporters” consistently highlighted proton and ion transport dysregulation, supporting the FPV framework while elucidating complex multi-protein transport assemblies integral to cancer survival and therapy resistance. Literature focusing on carbonic anhydrase isoforms (9–11, 50) underscored their critical role as key enzymatic regulators driving extracellular acidification and intracellular alkalinization, thus confirming their central position within the identified FPVs.

Additionally, systematic examination of metalloproteases within the TME illuminated their essential roles in extracellular matrix remodeling, invasion, and immune modulation (62–65). Notably, emerging data suggests copper’s indirect but pivotal role in regulating matrix metalloproteinase (MMP) activity through redox modulation and oxidative stress pathways (66, 67), reinforcing copper metabolism dysregulation as an integrative and actionable FPV.

The refined FPVs not only clarify core physiological vulnerabilities within the TME but also provide a coherent translational framework. This enabled the structured alignment of each FPV to corresponding intervention strategies involving Eigen/Zundel Complexes-Rich Water (EZC-Rich Water), facilitating hypothesis-driven research into its therapeutic potential.

Recent cutting-edge evidence positions copper metabolism as a critical nexus in cancer bioenergetics (68–70), notably influencing mitochondrial respiration and regulated cell death pathways through modulation of enzymes like cytochrome c oxidase (CcO)—the terminal complex of the mitochondrial electron transport chain, responsible for facilitating the transfer of electrons to oxygen and contributing directly to the generation of proton motive force for ATP synthesis (71). Intriguingly, studies (72–74) indicate that CcO activity may be enhanced by local availability of protonated water clusters (Eigen/Zundel configurations), suggesting that targeted supplementation with EZC-Rich Water could exploit copper-dependent metabolic vulnerabilities to selectively induce regulated cell death mechanisms such as cuproptosis (58) and ferroptosis (75).

In addition to the identification of FPVs, WP2 also aimed to extract and validate Critical Success Factors (CSF’s) necessary for effective modulation of the tumor microenvironment (TME). Using a multicriteria decision-support framework adapted from Bana e Costa et al. (1999) (24), the selected FPVs were systematically confronted with biological and physicochemical variables identified in the literature, prioritizing those factors capable of influencing pH dynamics, mitochondrial function, and membrane stability.

A total of eight condensed CSF’s were validated, each representing a key biological mechanisms implicated in tumor progression and potential therapeutic modulation via proton dynamics. These CSF’s were categorized based on their functional alignment with the FPV’s previously identified, thus consolidating a structured translational framework linking the acid-base, mitochondrial, and electrochemical dysregulation observed in cancer to possible intervention targets. The validated CSF’s are summarized in **Table 2**.

**Table 2:** Validated Critical Success Factors (CSF’s) and their association with Fundamental Points of View (FPV’s).

CSF	Description	Associated FPV’s	References
1. Proton-Dependent Enzymatic and Channels Regulation	Proton-dependent regulation of enzymatic and channel systems—including aquaporins, carbonic anhydrases, V-ATPases, Kir potassium channels, and Ca²^+^/calmodulin-dependent kinases (e.g., CaMKK2)—is central to controlling intracellular/extracellular proton gradients, membrane potential, and ionic homeostasis. This modulation directly influences metabolic signaling and bioenergetics within the TME.	FPV1, FPV2, FPV5	Kalinin et al. (2021); Rezuchova et al. (2023); Becker & Deitmer (2021); Fecikova et al. (2024); Verkman (2008); Verkman et al. (2013); Xia et al. (2023); Huang et al., 2021; Zhan et al. (2021); Angeli et al. (2020); Pastorek & Pastorekova (2015) (9–11, 50, 76–82)
2. Mitochondrial Bioenergetic Reprogramming	Dysregulation of oxidative phosphorylation, proton-coupled electron transport, and ATP synthesis with emphasis on cytochrome c oxidase-mediated redox modulation and copper-dependent processes.	FPV3, FPV5	Li et al. (2024); Di Gregorio et al. (2022); Wang et al. (2023); Cardone et al. (2023); Ruiz et al. (2021); Swaminathan & Gohil (2022); Supekar et al. (2016) (17–19, 69–72)
3. Ferroptosis and Iron-Dependent Cell Death Pathways	Iron-driven, lipid-peroxide-mediated cell death mechanisms shaping TME adaptation and therapeutic vulnerabilities.	FPV5	Wu et al. (2024); Lei at al (2024).; Wang et al. (2023); Yu et al. (2025) (75, 83–85)
4. Cuproptosis/Cuproplasia (Copper-Driven Cell Fate Modulation)	Copper-induced modulation of cell death and proliferation, mediated by copper-dependent enzymes and redox signaling pathways, directly modulated by ECZ water supplementation.	FPV5	Lu et al. (2025); Wang et al. (2023), Ge et al. (2022); Wu et al. (2024) (26, 58, 68, 75)
5. Metalloallostery and Metal Ion Signaling	Dynamic modulation of protein function, cell signaling, and plasticity driven by transition metals, notably copper and zinc, modulating TME response and cellular adaptation.	FPV5	Peters et al. (2019); Turunen et al. (2017); Wang et al. (2022); Ge et al. (2022); Gonzalez-Avila et al. (2020); Niland, et al. (2021). (63–65, 68, 86, 87)
6. Surface Charge-Driven Modulation of Cellular Interface and Ionic Permeability	Regulation of surface charge distribution (zeta potential), membrane electropermeability, and transmembrane ion/gas gradients through physicochemical modulation. This mechanism governs cellular uptake, intercellular signaling, and tumor-selective bioactive delivery, particularly enhanced by proton-dense molecular systems such as ECZ Water-derived hydrated proton complexes.	FPV2, FPV4	Fecikova et al. (2024); Miranda-Gonçalves et al. (2016); Schniers et al. (2021); Kim et al. (2025); Mendivil-Alvarado et al. (2023); Hughes (2024); Zhang et al. (2020) (50–53, 88–90)
7. Extracellular Vesicle-Mediated Communication	Vesicle-driven transport mechanisms of ions, metabolites, proteins, and nucleic acids, modulating pH, redox balance, and immune responses in the TME.	FPV1, FPV4	Gaspary et al. (2020); Parayath et al. (2020); Gondaliya et al. (2023); Semeradtova et al. (2025) (15, 91–93)
8. ROS and Redox Homeostasis Dysregulation	Imbalance between ROS production and antioxidant defenses leading to genomic instability, altered metabolism, and TME adaptation, with possible modulation via mitochondrial electron transport chain enzymes including CcO.	FPV3, FPV5	Scharping et al. (2021); Li et al. (2022); Rahman et al. (2023), Rhen et al. (2023) (94–97)

All the CSF’s identified and incorporated into our translational framework are grounded in a robust and up-to-date scientific literature. Most of these mechanisms—such as ion channel regulation, mitochondrial dysfunction, and redox imbalance—are widely recognized as consolidated drivers of tumor microenvironment (TME) dysregulation, with extensive experimental and clinical validation (98, 99).

It is important to note, however, that the inclusion of copper-driven metalloallostery as a CSF reflects a rapidly evolving understanding of cancer biology. Although this concept is relatively recent, it has gained considerable scientific traction as a key modulator of metabolic and signaling plasticity in cancer, as highlighted in recent reviews (26, 58, 68, 75). Its integration into our framework is intended to capture this emerging biological dimension, which is likely to shape future therapeutic strategies targeting TME vulnerabilities.

Likewise, the explicit integration of cytochrome c oxidase (CcO) activity within the CSF of mitochondrial bioenergetic reprogramming is supported by a growing body of evidence underscoring (72–74, 100) its pivotal role in mitochondrial respiration (71), cell death regulation (101, 102), and copper metabolism (68). CcO thus serves both as a mechanistic link and a potential therapeutic target within the TME.

In summary, WP2 provided a robust and integrative characterization of acid-base dysregulation, mitochondrial bioenergetic dysfunction, and metal homeostasis alterations within the tumor microenvironment. While most identified CSF’s are already well-established in cancer biology, this framework deliberately incorporates emerging yet scientifically substantiated mechanisms, thus positioning the research at the frontier of translational innovation. The systematic synthesis and formalization of FPVs and CSFs presented here offer a comprehensive foundation for assessing the therapeutic potential of EZC-Rich Water supplementation, clearly identifying actionable physiological targets for next-generation oncology interventions.

### 3.2 WP3 – translational potential of hydrated proton complexes and structured water chemistry for tumor microenvironment modulation

Considering the critical vulnerabilities identified in WP2—namely reversed pH gradients (FPV1), dysregulated proton and ion transport (FPV2), mitochondrial coupling dysfunction (FPV3), membrane electrochemical instability (FPV4), and alterations in metal ion homeostasis, particularly copper metabolism (FPV5)—WP3 was designed to explore physicochemical strategies capable of selectively addressing these targets through hydrated proton complexes. Hydrated proton complexes, specifically Eigen (H_9_O_4_^+^) and Zundel (H_5_O_2_^+^) clusters, emerged as promising translational candidates due to their distinctive capacity to modulate localized proton dynamics, stabilize membrane interactions, and maintain electrochemical balance without inducing systemic acidification or electrolyte disturbances (25, 28, 29). Therefore, WP3 aimed to systematically investigate the structural, biophysical, and translational properties of these proton complexes, emphasizing their unique solvation structures, enhanced proton mobility, and controlled interactions at biological membrane interfaces, in contrast to conventional diluted acid solutions containing free hydrogen ions (H^+^). Unlike conventional acid-based approaches, these protonated water clusters exert influence at the intersection of biophysical integrity, electrochemical signaling, and mitochondrial metabolism—thereby enabling a multidimensional modulation of tumor physiology.

Scientific literature indicates that proton conduction through water occurs predominantly via the Grotthuss mechanism, where protons are rapidly transferred along hydrogen-bonded water networks through a series of concerted molecular rearrangements (26, 27). Eigen and Zundel complexes are transient intermediates of this mechanism, facilitating highly mobile and directionally coordinated proton transfer, crucial for maintaining localized pH gradients in biological systems.

The translational relevance of hydrated proton complexes is rooted in three key properties. First, the enhanced proton mobility enabled by structured hydration promotes superior charge transport compared to free protons, allowing effective modulation of localized pH gradients without inducing abrupt systemic acidification (25, 29). Second, hydrated proton clusters exhibit high membrane interface stability, favorably interacting with biological membranes and modulating electrochemical gradients essential for mitochondrial bioenergetics and cellular signaling (103, 104). Third, their metastable organization reduces cytotoxic potential by minimizing non-specific acidification events and preserving electrolyte homeostasis, an important safety advantage for translational applications (28).

Recent advances have highlighted the translational impact of hydrated proton complexes, particularly in the context of copper metabolism within tumor cells. Copper acts as a vital cofactor for cytochrome c oxidase (CcO)—the terminal enzyme of the mitochondrial respiratory chain—which orchestrates ATP synthesis, redox homeostasis, and cell fate decisions. In tumors, where copper dysregulation and mitochondrial suppression are often observed, CcO activity is highly dependent on local proton availability, particularly through Eigen and Zundel cluster dynamics at its catalytic interface (72).

Thus, supplementation with EZC-Rich Water may synergize with the copper-dependent machinery of cancer cell mitochondria, restoring or enhancing CcO function and potentially tipping the balance towards increased mitochondrial activity, redox regulation, and induction of autophagy or cell death in metabolically vulnerable tumors. This convergence of proton transport, copper metabolism, and mitochondrial regulation provides a strong rationale for targeting the TME with strategies that bridge physical chemistry and metabolic oncology.

These findings provide a robust physicochemical basis for proposing Eigen/Zundel Complexes-Rich Water (EZC-Rich Water) as a clinically adaptable supplementation strategy aimed at reprogramming the tumor microenvironment. Instead of acting through systemic pH depression, EZC-Rich Water is hypothesized to promote localized proton enrichment at membrane interfaces and interstitial spaces, potentially stabilizing mitochondrial proton motive force, optimizing ATP synthesis, and restoring peritumoral electrochemical balance. A synthesis of these translational insights is presented in **Table 3**.

**Table 3:** Translational properties of Eigen/Zundel complexes relevant to tumor microenvironment modulation.

Property	Description	Implications for Tumor Microenvironment (TME)	References
Grotthuss Proton Transfer	Proton hopping via hydrated hydrogen-bonded networks.	Enables rapid and efficient localized pH modulation without systemic acidification.	Knight & Voth (2012); Popov et al. (2023); Markovitch et al. (2008); Intharathep et al. (2006); Soniat et al. (2015); Schröder et al. (2022). (26, 27, 105–108)
Structured Hydration Stability	Transient stabilization of hydrated proton clusters (Eigen, Zundel forms).	Facilitates controlled proton release and buffering at cellular interfaces.	Decka et al. (2015); Calio et al. (2021) (25, 29)
Membrane Interaction Capability	Favorable adsorption and influence on membrane potential and ion channels.	Supports mitochondrial bioenergetics and stabilization of membrane electrochemical dynamics.	Gabriel et al. (1996a); Gabriel et al. (1996b) (103, 104)
Low Cytotoxic Potential	Reduces risk of electrolyte imbalance and nonspecific acid stress.	Enhances safety profile for potential clinical translation.	Das et al. (2020) (28)

This approach is conceptually aligned with recent advances in physical oncology and nanomedicine, which recognize the tumor microenvironment’s electrochemical and mechanical aberrancies as therapeutic entry points. As reviewed by Nicolas-Boluda et al. (2018) (109), nanomaterials capable of modulating surface charge, tissue stiffness, and interstitial conductivity are increasingly explored to overcome drug resistance and enhance therapeutic penetration — a framework within which ECZ Water, rich in protonated water clusters, may function as a tunable, bioactive nano-agent.

Collectively, these findings establish a compelling physicochemical and translational rationale for the use of EZC-Rich Water in oncology. By enabling localized proton enrichment without systemic acid-base disruption, this strategy aims to restore peritumoral electrochemical stability, re-enable mitochondrial respiratory activity, and selectively sensitize metabolically vulnerable tumors to therapeutic modulation. Building on the physicochemical rationale presented above, WP4 systematically investigates the evidence supporting the translational use of EZC-Rich Water, with particular attention to its biomedical differentiation from conventional acidic solutions.

### 3.3 WP4 – evidence synthesis on EZC-Rich water supplementation

In continuity with the foundational FPVs mapped in WP2, WP4 reexamines their relationship with hydrated proton complexes under a translational lens and WP4 was initiated to systematically investigate the translational relevance of structured water supplementation enriched with hydrated proton complexes, specifically Eigen (H_9_O_4_^+^) and Zundel (H_5_O_2_^+^) forms. A systematic literature review (SLR) was registered under PROSPERO (CRD420251022205), following PRISMA guidelines (32), to address the central research question: Could EZC-Rich Water exhibit therapeutic potential, particularly in modulating the cellular microenvironment and inducing transient systemic pH variations?

The initial step involved defining the specific physicochemical properties of EZC-Rich Water and establishing a clear differentiation from conventional acidified water, forming the basis for subsequent multi-criteria analyses. **Table 4** presents a detailed comparative analysis.

**Table 4:** Comparison between Eigen/Zundel complex-rich water and diluted acid water.

Description	EZC-Rich Water	Diluted Acid Water (Haynes, 2014) (110)
Molecular Structure and Ion Composition	Composed of highly hydrated hydronium ions, primarily Eigen and Zundel complexes, enabling metastable contact ion pair formation. Facilitates distinct proton solvation compared to free ions (26, 27, 105–108).	Consists of free hydrogen ions (H^+^) and parent acid anions (e.g., Cl^-^ from HCl), with simpler, less structured hydration.
Electrochemical Properties	Demonstrates high electrical conductivity via Grotthuss mechanism proton transfer. (26, 105, 106, 108)	Exhibits electrical conductivity based on free ion mobility; proton transport is less efficient and organized.
Biological Interactions	Enhances protonation processes, compartmentalized proton regulation, and supports mitochondrial bioenergetics and pH homeostasis (25, 29, 106, 108, 111–113).	Alters systemic pH non-specifically; introduces potential side effects via anionic species.
Stability and Application	Requires controlled conditions to maintain hydrated proton integrity; gradual proton release upon ingestion favors smooth pH modulation (25, 28, 114–117).	More stable at environmental conditions but with non-specific pH impact and higher disruption risk.
Health Effects	Allows precise proton-mediated regulation with minimal electrolyte disturbance, reduced cytotoxicity risk (28, 29, 118).	Risks gastrointestinal irritation, mucosal damage, and electrolyte imbalances due to free acid content.

EZC-Rich Water can be synthesized through electrochemical methods. According to Alkhadra et al. (2022) (119), water electrolysis using direct current (DC) across ion-exchange membranes such as Nafion generates both hydronium (H_3_O^+^) and hydroxide (OH^-^) ions. The highly dynamic proton mobility via the Grotthuss mechanism poses challenges for stabilization, requiring immediate consumption after generation or low-temperature synthesis techniques to preserve the structured hydrated proton integrity (Markovitch et al., 2008 (105); Knight & Voth, 2012 (26); Schröder et al., 2022 (108)).

Alternative strategies, such as low-temperature chemical synthesis (Wurzbarger, 1986) (114), aim to extend the lifetime of hydrated proton complexes, potentially improving the translational viability of EZC-Rich Water for biomedical applications. Moreover, administration routes—including oral ingestion, transdermal absorption, or intravenous delivery—are under consideration to optimize proton gradient modulation for therapeutic effect without systemic acid-base disturbances.

To ensure conceptual coherence between the vulnerabilities mapped in the tumor microenvironment (FPVs identified in WP2) and the hypothesized modulatory actions of Eigen/Zundel Complexes-Rich Water, a comparative framework was developed to align each fundamental physiological target with its corresponding mechanism of intervention. This approach clarifies how the proposed supplementation may directly address each core axis of tumor dysregulation (see **Table 5**). By mapping one-to-one (or one-to-many) relationships, the framework underscores the translational logic of the strategy and offers a transparent rationale for the therapeutic potential of EZC-Rich Water in oncology.

**Table 5:** Comparative mapping table: alignment of tumor microenvironment FPVs with modulatory actions of EZC-rich water.

TME Vulnerability (WP2 FPV’s)	EZC-Rich Water Modulatory Actions	Mechanistic Effect	Key References
1. Reversed pH Gradient (Extracellular Acidification & Intracellular Alkalinization)	Localized Proton Gradient Modulation and Enhanced Proton Mobility via Grotthuss Mechanism	Restores physiological extracellular pH and corrects intracellular alkalinization by enabling controlled proton influx and efflux through structured hydration.	Persi et al. (2018); Shirmanova et al. (2015); Decka et al. (2015); Calio et al. (2021) (3, 4, 25, 29)
2. Dysregulated Proton/Ion Transport	Stabilization of Membrane Interfaces; Improved Membrane Electrochemical Integrity	Stabilizes ion and proton transport across membrane channels and transporters, restoring electrochemical gradients essential for cellular homeostasis.	Das et al. (2020); Verkman et al. (2013); Angeli et al. (2020) (28, 77, 81)
3. Mitochondrial Coupling Dysfunction	Optimization of Proton Motive Force and Bioenergetic Efficiency	Enhances mitochondrial oxidative phosphorylation efficiency by stabilizing localized proton availability, restoring mitochondrial ATP synthesis and reducing pathological ROS production.	Supekar et al. (2016); Dudev et al. (2015); Jastroch et al. (2010); Berry et al. (2018) (72, 120–122)
4. Membrane Electrochemical Instability	Regulation and Stabilization of Zeta Potential at Cellular Interfaces	Improves membrane integrity, reduces ion and gas leakage, and optimizes cellular communication and nutrient transport via electrochemical stabilization.	Boedtkjer & Pedersen (2020); Mallick & Agmon (2025); Zhu (2023) (35, 123, 124)
5. Metal Ion Homeostasis and Copper Metabolism Dysregulation	Regulation of Copper-Dependent Metabolic Pathways (e.g., Cytochrome c Oxidase Activity)	Restores copper-dependent enzymatic activity (e.g., cytochrome c oxidase), promoting redox balance, regulated cell death (cuproptosis/ferroptosis), and reduced tumor metabolic plasticity.	Ge et al. (2022); Supekar et al. (2016); Guo et al. (2025); Zhang et al. (2024) (68, 72, 125, 126)

To strengthen the translational foundation of the proposed intervention, WP4 reexamined the Critical Success Factors (CSF’s) identified in WP2 in light of the physicochemical properties of EZC-Rich Water. This comparative analysis demonstrates how hydrated proton complexes may interact with and modulate the core biological mechanisms represented by each CSF. **Table 6** presents this functional alignment, highlighting the potential contributions of EZC-Rich Water across distinct dimensions of tumor microenvironment dysregulation.

**Table 6:** Critical Success Factors (CSF’s) and potential contributions of EZC-rich water.

TME Vulnerability (WP2 CSF’s)	Description	Analysis of EZC-Rich Water Influence	Key References
Proton-Dependent Enzymatic and Channels Regulation	Regulation of intracellular/extracellular proton dynamics, ion and gas transport via proton-dependent enzymes and channels.	Facilitates controlled local proton enrichment, stabilizing enzyme activities (CAIX, V-ATPases) and ion-channel functionality, restoring normal pH gradients.	Verkman et al. (2013); Angeli et al. (2020); Becker (2019); Chen et al. (2024) (77, 81, 127, 128)
Mitochondrial Bioenergetic Reprogramming	Enhanced oxidative phosphorylation efficiency, redox balance, and copper-dependent enzyme activation, particularly cytochrome c oxidase (CcO).	Optimizes mitochondrial proton motive force, enhancing ATP production and reducing pathological ROS generation.	Supekar et al. (2016); Zhang et al. (2024); Zong et al. (2024); Nirody et al. (2020) (72, 126, 129, 130)
Ferroptosis and Iron-Dependent Cell Death Pathways	Iron-mediated, lipid peroxidation-driven cell death mechanism contributing to tumor progression and metabolic adaptability.	By modulating localized proton concentrations and supporting electrochemical balance, EZC-Rich Water may influence iron-dependent redox dynamics, potentially enhancing selective susceptibility to ferroptosis in metabolically stressed tumor cells.	Wang et al. (2023); Clemente et al. (2020); Berndt et al. (2024); Agmon et al. (2018); Roeck et al. (2025); Talachutla et al. (2021) (84, 131–135)
Cuproptosis/Cuproplasia (Copper-Driven Cell Fate Modulation)	Copper-induced modulation of cell survival and proliferation pathways mediated by redox-sensitive enzymes and metabolic signaling.	Provides structured proton support to copper-dependent pathways, selectively restoring or disrupting mitochondrial and cellular redox dynamics.	Wang (2024); Guan et al. (2024) (136, 137)
Metalloallostery and Metal Ion Signaling	Transition metal-mediated regulation of protein function, signal transduction, and cellular plasticity within the TME.	Supports optimal local proton concentrations for metal-dependent signaling, stabilizing metalloprotein structure/function, and enhancing redox signaling efficiency.	Gonzalez-Avila et al. (2020); Ge et al. (2022); Shen et al. (2023); Zhao et al. (2025); You et al. (2025) (68, 86, 138–140)
Surface Charge-Driven Modulation of Cellular Interface and Ionic Permeability	Regulation of membrane integrity, ion/gas leakage prevention, and modulation of surface charge (zeta potential) at membrane interfaces.	Stabilizes cellular zeta potential, improving membrane electrochemical integrity, nutrient absorption, and intercellular communication.	Hughes (2024); Mallick & (Agmon (2025); Agmon et al. (2018); Jin et al. (2021); Tyrode et al. (2020); Deplazes et al. (2018) (89, 123, 132, 141–143)
Extracellular Vesicle-Mediated Communication	Vesicle-driven transfer of metabolites, ions, nucleic acids, and proteins, modulating TME pH and redox balance.	Enhances vesicle-mediated proton transport efficiency, selectively modulating extracellular pH and intercellular signaling dynamics.	Parayath et al. (2020); Gondaliya et al. (2023); Semeradtova et al. (2025); Wu et al. (2021) (91–93, 144)
ROS and Redox Homeostasis Dysregulation	Imbalance between ROS generation and antioxidant mechanisms leading to genomic instability and metabolic adaptation.	Provides targeted proton regulation to optimize redox signaling pathways and minimize uncontrolled ROS generation.	Silverstein et al. (2021); Talachutla et al. (2021); Arntsen et al. (2021) (113, 135, 145);

The refined alignment of FPVs and CSFs further highlights the innovative translational potential of EZC-Rich Water, establishing a clear and mechanistically sound pathway for subsequent preclinical and clinical validation studies in cancer bioenergetics. This integrative matrix demonstrates that EZC-Rich Water acts simultaneously on multiple physiological targets linked to cancer progression. By delivering hydrated proton complexes capable of modulating membrane dynamics, buffering pH gradients, and enhancing mitochondrial proton motive force, this supplementation strategy presents a mechanistically grounded and translationally feasible intervention.

**Figure 4:**
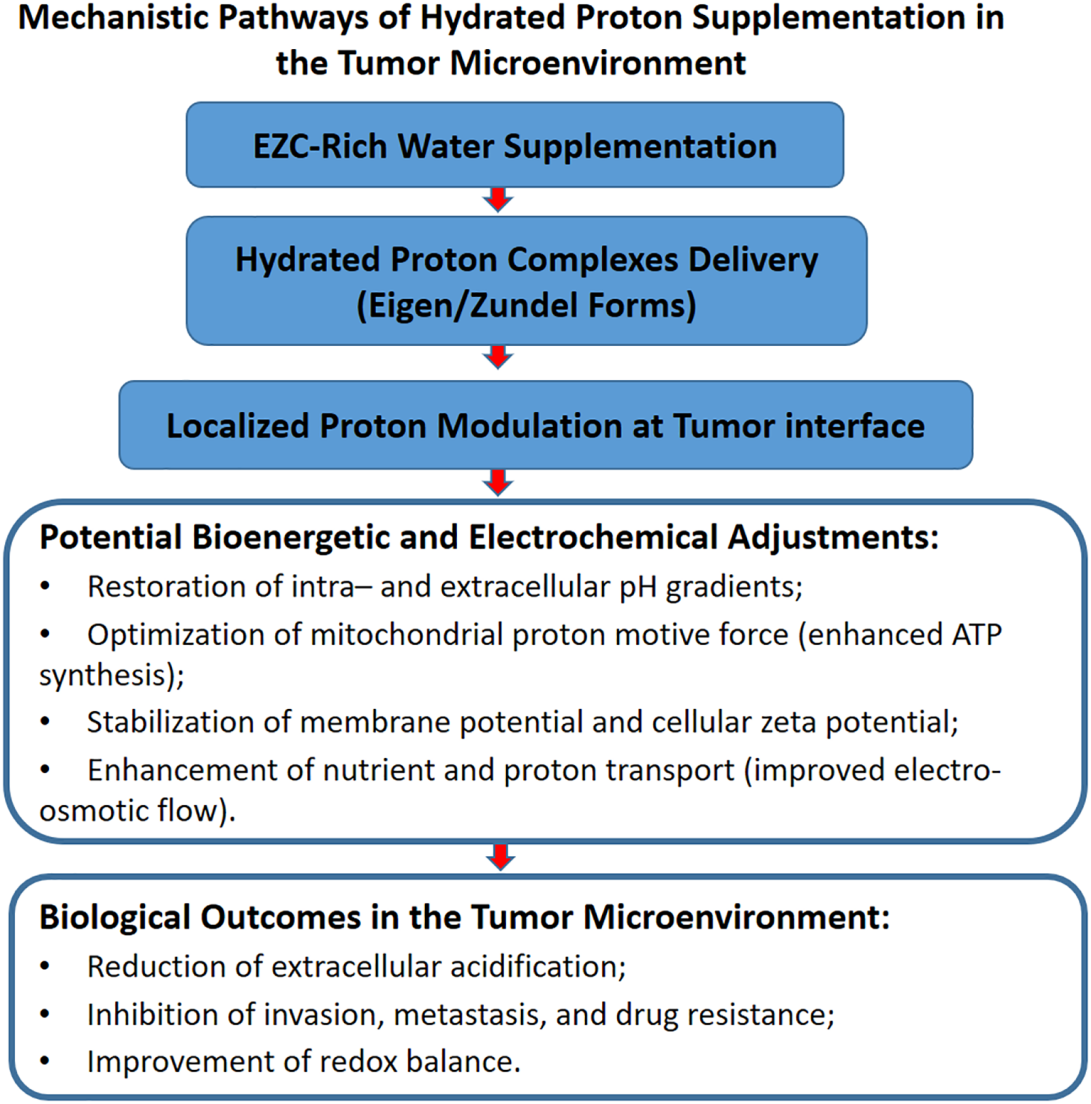
Structured supplementation with EZC-Rich Water facilitates targeted modulation of tumor microenvironment pH and bioenergetics, driving therapeutic outcomes.

While the refined alignment of FPVs and CSFs underscores the mechanistic rationale and translational feasibility of EZC-Rich Water, it is important to acknowledge that much of the current evidence supporting its potential derives from indirect experimental models, physicochemical simulations, and foundational studies in bioenergetics and interfacial proton dynamics. Direct validation of hydrated proton complexes in clinical or biomedical laboratory settings remains limited, reflecting the novelty of this approach rather than a lack of scientific plausibility. As such, the framework proposed here should be viewed as a theoretically robust and mechanistically informed starting point, warranting targeted experimental efforts to substantiate its therapeutic applications in cancer and beyond. Together with the conceptual synthesis illustrated in **Figure 4**, this framework positions EZC-Rich Water supplementation as a biophysical strategy with clinical relevance, potentially applicable in oncology and other bioenergetic dysfunction scenarios.

Analysis of the selected studies revealed that EZC-Rich Water supplementation exerts measurable impacts on five key FPVs, reinforcing its translational potential. First, it promotes modulation of intra- and extracellular pH gradients, facilitating the reversion of pathological pH inversion commonly observed in tumor microenvironments. Second, it optimizes mitochondrial ATP synthesis by enhancing proton motive force and improving overall energy conversion efficiency. Third, it regulates cellular membrane permeability, favoring more efficient nutrient transport, ion flux regulation, and stabilization of electrochemical gradients. Fourth, it enhances electrosmotic flow, supporting proton and molecule transport dynamics within tissue microenvironments. Finally, EZC-Rich Water stabilizes cellular zeta potential, contributing to the maintenance of membrane integrity and the optimization of cellular communication processes.

In addition to functional evidence, chemical analysis of EZC-Rich Water demonstrated its physicochemical differentiation from conventional diluted acidic solutions. Unlike free hydrogen ion-containing waters, EZC-Rich Water retains a metastable, structured organization of hydrated proton clusters (Eigen and Zundel complexes), promoting selective and regulated proton interactions without introducing disruptive ionic loads (25, 29, 105).

Thus, WP4 provided a solid translational foundation, demonstrating that EZC-Rich Water possesses distinct physicochemical and biological properties capable of addressing key bioenergetic vulnerabilities within the tumor microenvironment. Collectively, these findings underscore EZC-Rich Water’s innovative translational potential as a targeted therapeutic adjunct, and clearly justify subsequent experimental validation—ranging from preclinical models to eventual clinical trials—to establish its efficacy and safety profile in oncological applications. Beyond its oncological relevance, the experimental insights gained in WP4 also laid the groundwork for advancing hydrated proton supplementation from a theoretical construct into a broader translational research pathway, opening new perspectives for adjunctive interventions in metabolic disorders and mitochondrial dysfunction syndromes. These insights consolidate the translational foundation for future experimental validation and clinical exploration of EZC-Rich Water.

### 3.4 WP5 – integration of findings and formalization of translational hypothesis

Building on the mechanistic coherence established through WP2 to WP4, WP5 formalizes a structured translational hypothesis: that supplementation with Eigen/Zundel Complexes-Rich Water (EZC-Rich Water) may strategically modulate critical vulnerabilities within the tumor microenvironment (TME) by correcting localized pH gradients, enhancing mitochondrial bioenergetics, and stabilizing membrane electrochemical dynamics. Conceptually, this approach integrates recent advances in structured water chemistry, dielectric biology, and proton-mediated redox signaling, distinctly differing from conventional methods of systemic acidification or generalized antioxidant supplementation. Instead, EZC-Rich Water delivers metastable hydrated proton complexes (Eigen and Zundel forms), acting primarily through localized proton enrichment at cellular interfaces and mitochondrial membranes.

This translational proposition specifically addresses the FPVs previously identified as core vulnerabilities in the TME—reversed pH gradients, dysregulated ion transport, mitochondrial coupling dysfunction, membrane electrochemical instability, and metal ion homeostasis dysregulation. By providing a structured and controlled proton delivery mechanism, EZC-Rich Water may not only normalize aberrant extracellular acidification and intracellular alkalinization but also optimize mitochondrial proton gradients essential for ATP synthesis and redox balance. Moreover, this intervention could stabilize cellular membranes through modulation of zeta potential, further enhancing nutrient transport, intercellular communication, and overall metabolic resilience.

A critical component of this integrative hypothesis is the intersection between proton dynamics and copper metabolism. EZC-Rich Water supplementation may uniquely facilitate the optimal function of copper-dependent mitochondrial enzymes such as cytochrome c oxidase (CcO). By stabilizing localized proton availability at catalytic sites, EZC-Rich Water may enhance CcO activity, thus potentially triggering controlled mitochondrial reprogramming and regulated cell death pathways, including cuproptosis, autophagy, or ferroptosis (**Figure 4**).

To rigorously evaluate the translational feasibility and clinical viability of this proposal, we applied structured methodological criteria frameworks—SMART (Specific, Measurable, Achievable, Relevant, Time-bound) (33) and FINER (Feasible, Interesting, Novel, Ethical, Relevant) (34). This critical evaluation confirmed that EZC-Rich Water supplementation meets key parameters essential for advancing translational oncology research. **Table 7** summarizes this structured feasibility assessment, clearly outlining how the proposed therapeutic strategy satisfies essential scientific, practical, and ethical standards.

**Table 7:** Outcome of SMART and FINER evaluation for EZC-rich water supplementation hypothesis.

Criteria	Analysis
Specific (S)	Targets precise modulation of TME pH gradients, mitochondrial bioenergetics, and membrane electrochemical stability.
Measurable (M)	Quantifiable via biomarkers including intra-/extracellular pH, mitochondrial ATP output, membrane zeta potential, proton flux, and oxidative stress markers.
Achievable (A)	Current technology enables synthesis, stabilization, and delivery of structured hydrated proton complexes; experimental validation feasible with available *in vitro* and *in vivo* models.
Relevant (R)	Directly addresses unmet clinical needs in cancer bioenergetics, providing a safe, non-toxic strategy for metabolic reprogramming.
Time-bound (T)	Preclinical validation achievable within 12–24 months, supporting near-term translational advancement.
Feasible (F)	Methodologies for production, administration, and bioenergetic assessment of EZC-Rich Water are readily available and scalable.
Interesting (I)	Provides a fundamentally innovative biophysical approach with potential broad interdisciplinary impact across oncology, metabolism, and bioenergetics.
Novel (N)	Introduces a distinct therapeutic class based on structured hydrated proton supplementation, divergent from conventional approaches.
Ethical (E)	Aligns with ethical research standards, presenting a favorable anticipated safety profile due to the controlled physicochemical properties of hydrated proton complexes.
Relevant (R)	Highly pertinent to current clinical oncology and broader bioenergetic health challenges, addressing significant gaps in existing therapeutic strategies.

Collectively, WP5 affirms that the supplementation with EZC-Rich Water possesses substantial scientific credibility and practical feasibility. By clearly delineating the bioenergetic and physicochemical rationale, as well as the strategic alignment between Fundamental Points of View (FPV’s) and validated Critical Success Factors (CSF’s), this synthesis lays a solid translational foundation. Therefore, EZC-Rich Water represents a scientifically robust, ethically sound, and clinically relevant adjunctive intervention strategy, warranting targeted preclinical and clinical validation efforts in oncology and broader bioenergetic optimization contexts.

By integrating these mechanistic insights into a coherent translational framework, this study robustly supports the hypothesis that structured hydrated proton supplementation can act as a low-risk, multi-targeted therapeutic strategy, addressing multiple bioenergetic and metabolic vulnerabilities simultaneously. The comprehensive synthesis provided here lays a firm foundation for further exploration of clinical implications, translational relevance, and innovative therapeutic applications, as explored in the following discussion.

## 4 Discussion

This study presents EZC-Rich Water supplementation as a translational bioenergetic intervention grounded in the regulation of proton gradients and the restoration of acid–base and metabolic homeostasis within the tumor microenvironment, particularly targeting the Warburg effect and mitochondrial dysfunction. Although still in the early stages of clinical exploration, the mechanistic rationale and experimental integration proposed here provide a coherent foundation for targeted preclinical exploration. By bridging quantum-level proton dynamics with systemic physiological regulation, this approach introduces a new class of bioenergetic strategies that extend beyond traditional pharmacological or metabolic interventions. Quantum-level proton transport dynamics, referring to the coherent, rapid proton transfers via hydrogen-bonded water networks described by the Grotthuss mechanism, have emerging translational implications for biomedical interventions (27, 145–148). Recent studies reinforce the presence of Grotthuss-type proton transfer within biological systems, including active enzyme sites (Zlobin et al., 2024 (147)) and voltage-gated proton channels (Morgan & DeCoursey, 2003 (148)), suggesting that structured hydrated proton movement is not limited to bulk water but also plays a role in bioenergetic and redox regulation. These insights provide a strong molecular rationale for exploring translational applications of proton-rich supplementation strategies, such as EZC-Rich Water.

Hydrated proton complexes—Eigen and Zundel structures—possess a unique capacity for facilitating localized proton mobility via the Grotthuss mechanism. Their application in aqueous supplementation form enables compartmentalized acid-base modulation without systemic acidosis, preserving intracellular signaling fidelity, mitochondrial efficiency, and membrane integrity. These structured protons, when delivered via EZC-Rich Water, may act on key bioenergetic axes, modulating mitochondrial oxidative phosphorylation, supporting ATP synthesis, and reducing reactive oxygen species generation (Lee & Nandab, 2020 (149); Aklima et al., 2021 (150); Bertholet et al., 2022 (151)).

The evidence consolidated across the Work Packages suggests that transient and localized proton adjustments, as promoted by EZC-Rich Water, can positively influence zeta potential, membrane fluidity, redox resilience, and electrosmotic flow—all of which are severely impaired in cancerous tissues. These effects are mechanistically plausible and conceptually aligned with previous theoretical explorations regarding carbon dioxide’s hormetic potential in the tumor microenvironment. Previous research conducted by the same group (Gaspary et al., 2024 (15)) have demonstrated that CO_2_’s conversion to carbonic acid may assist in buffering extracellular acidity, potentially enhancing immunotherapeutic responses and modulating key enzymatic systems such as carbonic anhydrase IX (Ronca et al., 2024 (152)), heme oxygenase (Surh et al., 2020 (153)), and matrix metalloproteinases (Niland et al., 2021 (87)).

By integrating these perspectives, it becomes evident that modulating local proton dynamics—whether via CO_2_ pathways or via structured water supplementation—offers a convergent route to reprogram the biochemical landscape of malignant tissues. Both approaches—EZC-Rich Water and controlled CO_2_ exposure—appear capable of influencing critical elements of the tumor ecosystem, including pH regulation, mitochondrial metabolism, immune surveillance, and cellular communication.

The distinction between these two modalities lies primarily in delivery and control. EZC-Rich Water offers a targeted, non-gaseous, and potentially safer method of introducing hydrated protons in a metastable and bioavailable form. Meanwhile, CO_2_, though physiologically potent, poses challenges in delivery standardization and long-term safety, particularly under chronic or high-concentration exposure conditions (15). Nonetheless, the theoretical convergence highlights the growing recognition of proton gradients as dynamic modulators of cellular behavior and disease progression.

Despite these promising mechanistic foundations, it must be acknowledged that these hypotheses remain at an early experimental stage. Observational case reports from the AuBento Institute suggest preliminary clinical benefits following CO_2_-modulation protocols, such as PSA reduction and tumor regression (15); however, rigorous, controlled clinical trials are necessary to substantiate both mechanisms (Chesbrough, 2003 (22); Flessa & Huebner, 2021 (154). Additionally, the absence of adequate *in vitro* models capable of replicating systemic adaptive processes highlights the complexity inherent in studying such bioenergetic interventions.

From a translational standpoint, one of the most pressing challenges is the refinement of dosing protocols, definition of optimal proton concentrations, and assessment of long-term systemic impacts. Whether administered orally, transdermally, or intravenously, EZC-Rich Water demands comprehensive profiling of bioavailability, pharmacokinetics, systemic distribution, and potential toxicity under standardized preclinical conditions. These evaluations must also consider compatibility with existing therapies and establish stable, replicable dosage regimens through longitudinal studies. Similarly, CO_2_-based interventions must delineate precise thresholds between beneficial hormetic exposure and harmful systemic acidosis, while identifying reliable biomarkers of therapeutic response and physiological resilience (15). Despite the robust theoretical rationale developed thus far, clinical applicability remains speculative in the absence of dose standardization, physicochemical stability assessments, and safety data in human models. These are not minor concerns, but critical gatekeepers for advancing toward empirical validation. As such, rigorously designed experimental studies are imperative to move beyond exploratory plausibility and establish a solid foundation for clinical translation.

The unifying principle behind both strategies is the recognition that precise modulation of proton gradients—rather than direct cellular cytotoxicity—may represent a fundamentally new paradigm in oncology and bioenergetic medicine. Instead of targeting the tumor directly, the approach focuses on reprogramming the biological terrain to create conditions unfavorable for malignancy and conducive to systemic resilience.

Emerging evidence strongly supports the pivotal role of proton fluxes in mitochondrial bioenergetics, oxidative phosphorylation efficiency, and redox homeostasis. Hydronium ions, inherently present in aqueous environments, play critical roles in intracellular signaling and energy metabolism. Localized adjustments in proton distribution, as hypothesized with EZC-Rich Water supplementation, could facilitate ion transport across membranes, stabilize mitochondrial function, and optimize redox balance, offering a finely tuned mechanism to enhance cellular adaptation and resilience (Lee & Nandab, 2020; Aklima et al., 2021; Bertholet et al., 2022) (149–151).

In support of this hypothesis, experimental findings indicate that hydronium ions can modulate phospholipid bilayer properties, impacting membrane conductivity, ion flux, and signal transduction (Deplazes et al., 2018) (143). These phenomena reinforce the concept that structured hydration-mediated proton delivery could exert tangible bioenergetic benefits at the membrane interface level.

Although WP5 provided a structured feasibility analysis of the proposed intervention using SMART and FINER frameworks, it also served to consolidate the key mechanistic dimensions supporting the clinical plausibility of EZC-Rich Water supplementation. Based on the evidence synthesized throughout the WPs, **Table 8** highlights how transient proton gradient modulation may influence key physiological systems across local, cellular, and systemic levels.

**Table 8:** Local, cellular, and therapeutic considerations of EZC-rich water supplementation.

Mechanisms & Considerations	Description	Supporting Evidence
Localized Antioxidant Activity	Hydronium-rich water may neutralize reactive oxygen species (ROS) at specific sites without significantly altering systemic pH.	Kwang-Hua (2019) (118)
Effects on Cellular Function	Enhances cell hydration and microenvironmental regulation, particularly around ion channels and proton transporters such as AQP1.	Tyrode et al. (2020); Riveros-Perez & Rivero (2017) (142, 155)
Immune Response Modulation	Localized hydronium presence may influence immune cell behavior, especially in the gastrointestinal tract, potentially modulating inflammation and immune defense.	Gabriel et al. (1996c) (156)
Therapeutic Potential Without Systemic pH Alteration	Structured proton hydration enables localized effects on redox and immune function without inducing systemic acid-base imbalance.	Mouhat et al. (2023) (117)
Safety and Efficacy	Requires experimental confirmation regarding safe dosage, long-term exposure effects, and optimal delivery routes (oral, transdermal, IV).	Hulley et al. (2007) (34)
Need for Scientific Validation	Robust preclinical and clinical research is necessary to confirm efficacy, safety, and reproducibility of biological effects.	Strand (2020); Hulley et al. (2007) (34, 157)

Taken together, the findings from WP1 to WP5 construct a robust translational framework supporting the therapeutic potential of EZC-Rich Water supplementation. By targeting the central dysfunctions of the tumor microenvironment — pH inversion, mitochondrial inefficiency, electrochemical instability — this approach introduces a novel, biophysically grounded, and biologically coherent adjunctive strategy. The integration of hydrated proton complexes within a clinically adaptable format, combined with their low cytotoxicity and precise biochemical targeting, advances the field of bioenergetic modulation in oncology.

**Figure 5:**
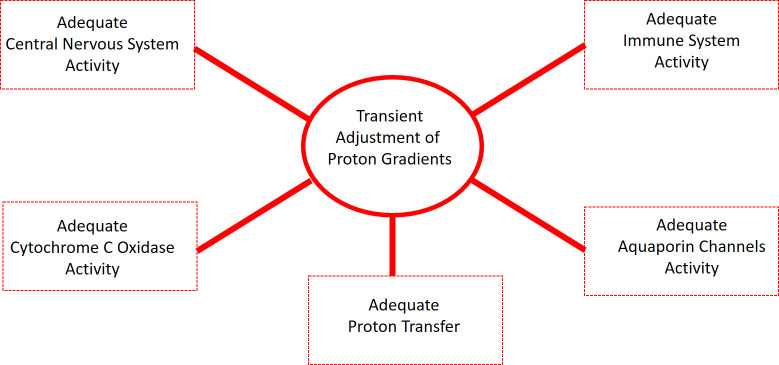
Conceptual representation of the systemic physiological outcomes mediated by the transient adjustment of proton gradients, central to the biological action of EZC-Rich Water supplementation.

These mechanisms are not exclusive to tumor regulation but probably extend to general homeostatic processes such as membrane stability, redox signaling, immune responses, and targeted pH buffering. This reinforces the hypothesis that hydrated proton supplementation may act not merely as an acidifying agent, but as a bioenergetic modulator with multidimensional therapeutic potential. As illustrated in **Figure 5**, the transient and localized adjustment of proton gradients — the mechanistic basis of the proposed intervention — may influence multiple physiological systems simultaneously. These include aquaporin channel regulation, cytochrome c oxidase activity, cellular immune responses, central nervous system homeostasis, and efficient proton transfer. This multi-domain responsiveness reinforces the broader systemic relevance of the intervention beyond cancer alone, with implications in neurobiology, immunology, and metabolic disease.

Interestingly, the mechanistic rationale behind EZC-Rich Water supplementation converges with other non-pharmacological strategies such as controlled CO_2_ exposure. Both approaches act by subtly influencing interfacial proton dynamics—particularly at membrane boundaries—affecting localized electrochemical conditions such as charge polarization and redox signaling, without inducing systemic acid-base disruption. This convergence reinforces the symbolic unity of the intervention: metabolic restoration through precise interfacial modulation rather than global biochemical interference, offering a conceptually elegant and minimally invasive approach to reprogram tumor metabolism.

The hypothesis built throughout the structured research stages now calls for a critical examination in light of current scientific literature. In the following section, we explore the implications, strengths, limitations, and future directions for hydrated proton supplementation — contextualizing its bioenergetic logic, its translational novelty, and its potential clinical applications in cancer therapy and beyond.

By consolidating these mechanisms into an integrative translational summary, this multifaceted and interdisciplinary research provides robust support for the hypothesis that hydrated proton supplementation could serve as a low-risk, multi-targeted strategy—particularly effective in reversing the Warburg effect, restoring mitochondrial function, and optimizing broader biological processes in both pathological and physiological contexts. These insights establish a solid foundation for broader reflections on implications, clinical relevance, and future research directions explored throughout the discussion. Moreover, while the current theoretical framework robustly outlines the translational potential of EZC-Rich Water supplementation in oncology, additional validation across multiple experimental models will be essential to ensure reproducibility and generalizability of the findings. Future studies should specifically consider randomized controlled trials designed to evaluate key bioenergetic and clinical biomarkers outlined in this research, such as mitochondrial ATP output, proton flux dynamics, membrane electrochemical stability, and redox homeostasis. Furthermore, extending the application of EZC-Rich Water supplementation beyond oncology to other metabolic, inflammatory, and neurodegenerative diseases could provide broader validation of its systemic therapeutic potential, reinforcing its interdisciplinary significance and enhancing its translational impact across diverse clinical domains.

These results also support the emergence of a novel therapeutic paradigm—one that integrates biophysical insight, metabolic precision, and physiological modulation. EZC-Rich Water may be representative of a new class of adjunctive strategies that operate at the intersection of structured hydration chemistry, mitochondrial bioenergetics, and tumor interface dynamics. As such, this supplementation model may transcend its immediate oncological scope and signal broader applications across regenerative, metabolic, and redox-centered medicine. Although this study is framed within the oncological landscape, the underlying principles of hydrated proton dynamics—particularly in mitochondrial function and membrane electrochemical stability—suggest translational potential in metabolic disorders, chronic inflammation, and neurodegenerative syndromes. These applications should be explored in parallel to preclinical validation in cancer models.

Ultimately, while the theoretical framework is well-grounded, translational credibility will only be achieved through rigorous experimental validation across preclinical and clinical models—ensuring reproducibility, safety, and real-world efficacy.

## 5 Conclusions

This study introduces Eigen/Zundel Complexes-Rich Water (EZC-Rich Water) as a mechanistically grounded and translationally feasible approach for tumor microenvironment (TME) modulation. By addressing proton gradient dysregulation—a central hallmark of cancer bioenergetics—this supplementation strategy offers a novel route to reprogram pathological pH inversion, mitochondrial dysfunction, and membrane electrochemical instability.

Unlike conventional acidified solutions, EZC-Rich Water leverages metastable hydrated proton clusters—Eigen (H_9_O_4_^+^) and Zundel (H_5_O_2+_)—to enhance proton mobility and interface stability without systemic acidosis. The integrative mapping between Fundamental Physiological Vulnerabilities (FPVs) and Critical Success Factors (CSFs) strengthens its therapeutic plausibility, particularly in contexts marked by the Warburg effect, ion transport imbalance, and copper-dependent mitochondrial suppression.

The findings also highlight mechanistic convergence with other bioenergetic interventions—such as controlled CO_2_ exposure—reinforcing the emerging importance of interfacial proton dynamics in translational oncology and metabolic research.

Despite its conceptual rigor, the clinical viability of EZC-Rich Water remains dependent on empirical validation. Key priorities for future research include: standardization of proton concentrations, stability profiling, pharmacokinetic evaluation, and safety assessment through preclinical and human studies. Randomized controlled trials should be designed to monitor specific biomarkers of mitochondrial function, redox modulation, and peritumoral pH regulation, providing a robust foundation for regulatory advancement.

In summary, EZC-Rich Water supplementation emerges as a promising adjunctive strategy within a new class of biophysically informed therapeutics. While current evidence supports its theoretical and mechanistic potential, its definitive translational value will depend on sustained interdisciplinary efforts and rigorous experimental corroboration. If validated, this intervention may contribute meaningfully to the evolving landscape of personalized and metabolically targeted medicine.

## Data Availability

The original contributions presented in the study are included in the article. Further inquiries can be directed to the corresponding author.
